# Epidemiological Characteristics of Pediatric Ocular Trauma in China: A Multicenter Retrospective Hospital-Based Study

**DOI:** 10.1155/2022/4847027

**Published:** 2022-07-06

**Authors:** Yaxin Zhang, Kang Feng, Hua Yan

**Affiliations:** ^1^Department of Ophthalmology, Tianjin Medical University General Hospital, Tianjin, China; ^2^Department of Ophthalmology, Peking University Third Hospital, Beijing, China; ^3^Tianjin Medical University, Tianjin, China

## Abstract

**Purpose:**

The objective of the study was to obtain the incidence characteristics of pediatric ocular trauma in mainland China and provide some feasible suggestions of prevention.

**Methods:**

The patients with pediatric ocular injuries, who were (≤14) hospitalized between January 1, 2019, and December 31, 2020, were included. Patient demographics (age and sex), injury natural characteristics (causes, site, and types), geographic location, and interval of hospital admission were analyzed.

**Results:**

A total of 13525 cases were identified, and 1973 (14.6%) occurred in children (male accounts 70.9%) with a mean (SD) age of 6.5 (3.4) years. Cases among minors in 2020 dropped by 8.3% compared to 2019, while the former vulnerability to eye injuries has been shifted from the outdoors to home (51.4%, 1015/1973). The main causes of injury are flying objects (31.9%), traffic injury (23.5%), and blunt injury (21.8%), which lead to the main types of injury such as penetrating injury (48.3%), rupture of the globe (18.1%), and contusion trauma (13.1%). If the VA after injury is above CF, the VA after discharge is more likely to be above CF (OR 18.3, 11.9–28.1; *p* < 0.001). Similarly, age (OR 2.0, 1.3–3.1; *p*=0.001) and intraocular pressure after injury (OR 0.9, 0.9–1.0; *p*=0.009) also affect the intraocular pressure at discharge.

**Conclusions:**

In mainland China, the main injury types are penetrating injury and rupture of the globe with home being the most dangerous place. Prevention strategies should focus on parents' education and protective measures indoors. Visual acuity after injury can be used as a predictor of visual acuity after treatment.

## 1. Introduction

Ocular trauma as the leading cause of monocular visual disability and noncongenital unilateral blindness in children [1], constitutes 7% of all bodily injuries and 10–15% of all eye diseases [2, 3], is responsible for up to 280,000 hospital admissions annually in children aged less than 15 years [4]. Minor eye trauma can lead to serious consequences, such as amblyopia and endophthalmitis [5, 6], and the affected individuals must learn to endure marked socioeconomic implications and burden [7, 8]. More seriously, it has a very serious negative impact on the growth of minors, such as the risk for delayed learning, limitations in skills acquisition, and impaired social relationships [9], leading to a lower quality-of-life score compared with their peers [10]. Public health service will also have to bear the serious consequences of trauma [11], mainly referring to the financial burden.

By fully understanding the characteristics of juvenile eye trauma, the constituent ratio in different situations can help clinicians and researchers better analyze the patient's condition and make the most effective treatment. Numerous studies have evaluated various aspects of pediatric ocular trauma in China, and some scholars have studied the epidemiological characteristics of ocular trauma in children in local areas [12, 13]; however, there is no comprehensive study based on national cases. This study was conducted to evaluate the epidemiologic patterns of ocular injuries in children of mainland China, using available data from 30 major tertiary hospitals from 2019 to 2020.

## 2. Materials and Methods

The study conformed to the Declaration of Helsinki and was approved by the Review Board/Ethics Committee of Tianjin Medical University.

A multicenter cross-sectional study was performed, which has been described in the preprint [14]. All patients who presented to the ophthalmology department and were admitted to the hospital from 30 hospitals in 2019 and 2020 (from 1^st^ January to 31^st^ December, respectively) were included in the study. Those victims who did not require to be hospitalized and were older than 14 years old were excluded.

According to the geographical characteristics of China, we divided the area into seven geographical regions (see Additional Information) in the study. We selected comprehensive tertiary A hospitals or eye specialist hospitals from 22 cities covering 20 provinces, municipalities, and autonomous regions in China as the target hospitals that were listed at the end of the article. All participating hospitals, which are all good at salvaging injured eyes, were from China Eye Injury Alliance. Demographic information and injured information of all cases were collected based on the hospitalized records, which were inputted into the electronic version of the Excel data frame. The research group summarized the data from the participating hospitals and contacted the registrars to retrieve the original case data if there were missing and incorrect records.

In this study, we analyzed demographic data including regions of victims, patients' sex and age, causes of injury, the site and the type of injury, and changes in visual acuity and intraocular pressure after injury and discharge.

Descriptive data were expressed as frequencies (percentage %). Binary logistic regressions as well as *χ*^2^ tests were conducted to identify age-adjusted and sex-adjusted predictors for VA and IOP after injury and discharge. All calculations were performed using SPSS version 26.0 statistical software (SPSS Inc, Chicago, Illinois, USA), and data collation and figures were implemented using Excel (2016 version, Microsoft, Redmond, USA) and GraphPad software 8.0.2 (GraphPad Software, Inc. San Diego, California, U.S.).

## 3. Results and Discussion

A total of 13,525 inpatient records were collected, including 1,973 cases of juvenile ocular trauma that were eligible for the study (1,029 cases in 2019 and 944 cases in 2020). In 2019, most of the cases were concentrated in central (*n* = 308, 29.9%), eastern (*n* = 241, 23.4%), and northern China (190, 18.5%). The distribution changed in 2020, with central China still having the highest number of cases among minors (*n* = 274, 29.0%), followed by north (*n* = 210, 22.2%) and southwest China (*n* = 156, 16.5%). Most of the male cases were concentrated in central (*n* = 416, 29.8%) China, while the number of cases is similar in north (*n* = 297, 21.2%), east (*n* = 262, 18.7%), and southwest (*n* = 226, 16.2%) China, with females having the same distribution (Table 1).

**Figure 1:**
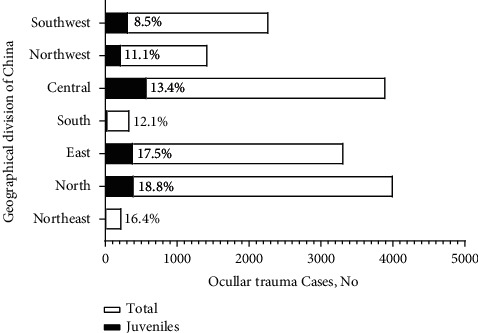
Proportion of pediatric eye trauma cases among all injured patients in different geographical regions in China.

In different geographical regions of China, the number of cases in north China, south China, and northwest China increased by 10.5%, 46.7%, and 27.3% (year-on-year), respectively, while the other four regions decreased by 50.0%, 37.8%, 11.0%, and 4.9%, respectively. The proportion of pediatric ocular trauma in each geographical region is shown in Figure 1. The average age of the patients was 6.5 (SD = 3.4) years, and the most common age of ocular trauma was 4 to 9 (*n* = 1139, 57.7%) years old (Table 2). Moreover, the injured children can be carried to a hospital within one day in most regions (*n* = 1525, 77.3%), but there are a few children (*n* = 186, 9.4%) who have been delayed for six days or more.

**Figure 2:**
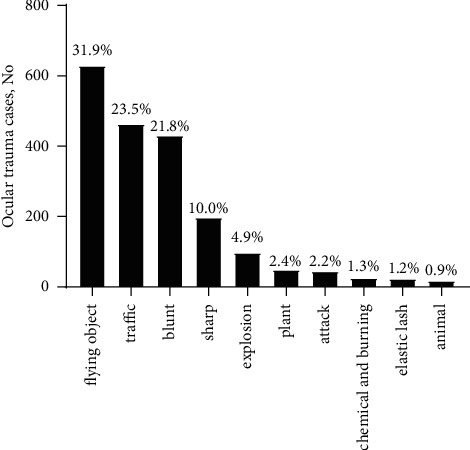
Distribution of pediatric ocular trauma caused by the type of injury from 2019 to 2020.

Flying objects (*n* = 318, 30.9% in 2019 and *n* = 311, 32.9% in 2020) were the leading cause of pediatric injury in both 2019 and 2020, followed by traffic injury (*n* = 255, 24.8% in 2019 and *n* = 208, 22.0% in 2020) and blunt injury (*n* = 218, 21.2% in 2019 and *n* = 212, 22.5% in 2020). The causes of injury specified are presented in Figure 2.

Home is still the main place of injury in children at any time (*n* = 516, 50.1% in 2019 and *n* = 499, 52.9% in 2020). Public buildings (*n* = 166, 16.1% in 2019 and *n* = 137, 14.5% in 2020) and fields (*n* = 159, 15.5% in 2019 and *n* = 102, 10.8% in 2020) are also places where minors are prone to occur eye trauma, even the number of cases decreased in 2020.

**Figure 3:**
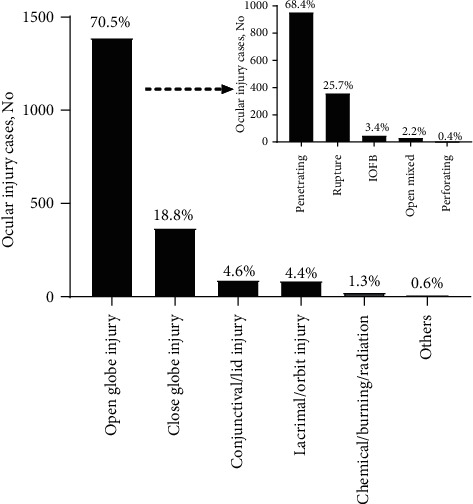
Details of pediatric ocular trauma caused by the type of injury from 2019 to 2020.

The main types of eye injuries in minors are still open globe injuries (*n* = 1391, 70.5%), in which the penetrating injury (*n* = 952, 68.4%) and globe rupture (*n* = 357, 25.7%) are the main types. Of all the penetrating injuries, 4 cases were suffered from endophthalmitis caused by flying objects at home. In children, nonmechanical eye injuries are rare, which only account for 25 cases in 2019 and 2020 (Figure 3).

**Figure 4:**
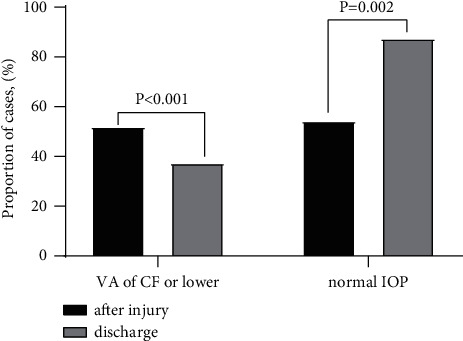
Comparison of visual acuity and intraocular pressure between after injury and discharge in the children with exact data.

The visual acuity (VA) and intraocular pressure (IOP) were changed at discharge. VA after injury was available in 984 cases (49.9%), among which 51.9% (511/984) of the children had VA of the count finger (CF) or lower after injury. At discharge (951 cases supplied VA, accounting for 48.2%), this proportion dropped to 37.4% (356/951, *χ*^2^ = 278.2, *p* < 0.001) (Figure 4).

The odds of having an unsatisfactory outcome (VA below CF) were significantly higher in those who had poor vision after injury (OR 18.3, 11.9–28.1; *p* < 0.001), while the type of trauma, surgeon's title, and surgical interval were not statistically significant. As to the IOP, we graded it into three categories (low : IOP<10; normal: 10 ≤ IOP ≤ 21; high: IOP>21). Of all the cases, reported IOP conditions (56.8% after injury and 66.8% discharge, respectively), among which only 54.2% children had a normal IOP after injury, and the proportion rose to 87.4% (*χ*^2^ = 9.5, *p*=0.002) after treatment (Figure 4). Likewise, the odds of having a normal value (10 ≤ IOP ≤ 21) were significantly higher in those who had a normal IOP after injury (OR 2.0, 1.3–3.1; *p*=0.001) and who were younger (OR 0.9, 0.9–1.0; *p*=0.009). The type of trauma, surgeon's title, and surgical interval were not statistically significant. In addition, evisceration combined with ocular prosthesis implantation was implemented in 37 cases (1.88%, 37/1973).

Since COVID-19 spread in China and around the world [15, 16], the frequency and sites of accidents causing severe ocular trauma have been affected [17]. For minors, campus life and activities outside have been greatly reduced, but the ocular injuries remained prevalent [18]. Compared with other countries [19], the present situation of children's eye trauma in mainland China is more serious (accounting for 14.6%, 1973/13525). Therefore, no matter what period society is, the prevention of minor eye trauma should not be ignored.

So far, little is known about the specific geographic and demographic characteristics of eye trauma in children, leading to destructive vision loss [20]. Hence, data from a country with a large population providing overview of the epidemiological characteristics of pediatric eye trauma were extremely essential. In China, parents tend to choose comprehensive tertiary A hospitals or eye specialist hospitals to seek medical attention for their children. And in China usually, the first-stage surgery is conducted at the primary hospitals and then in the higher-level comprehensive or specialized hospitals for the second-stage surgery [21, 22]. Therefore, we selected comprehensive tertiary A hospitals and eye specialist eye hospitals from the above 20 provinces, municipalities, and autonomous regions as the target hospitals that were listed at the end of the article, and all the participating hospitals covered all the seven regions of China.

Scholars always paid more attention to minor eye injuries, an important source of preventable monocular blindness [23], and it is still an issue deserving to be studied. As reported, the curiosity and reckless imitation combined with immature motor skills and limited general knowledge make juveniles more vulnerable to eye injuries [24]. When minors make dangerous attempts, probably out of fear, they often hide from their parents [25] and that is when tragedies happen. Consistent with other similar studies [26, 27], the male-to-female ratio is 2.4 : 1 in this study. Generally, boys are more likely to try aggressive and violent nature of activities than girls do and the curiosity drives them to try new and exciting activities which make males most of the pediatric eye trauma patients [28, 29].

Publications have shown that children's eye injuries occur at different ages, and one study revealed that the common age of ocular trauma in children is usually between 6 and 11 years old [30], while another study said in the age group of 5–10 years [28]. In our study, most patients come from 4 to 9 years old and the average age is 6.5 (SD = 3.4) years old. In view of China's national conditions, a considerable number of young children cannot be well cared, for their parents went out to work in urban, leaving these children as “left-behind children” in rural area. Therefore, compared with foreign countries, the incidence of juvenile eye trauma in China is more likely to occur in younger children.

Both in China and other countries, home is a common place for eye injuries among minors [28, 30, 31], given that minors themselves spend most of their time at home, especially during the COVID-19 period. Research also reported that household cleaners, children's toys incorporating projectiles, and sharp-edged domestic items are leading causes of vision-threatening burns or penetration of the globe [32]. Besides, parents generally relax their supervision of their children at home. Most of the time, minors are alone with relatively weak awareness of the identification of dangerous substances and body protection. When several children play together, trauma may be caused by flying objects (31.9%) as shown in this study. Minors have also suffered a high rate of injuries in public buildings, mainly in schools [33, 34], a site with students of similar ages. Although there are many teachers at school, accidents may occur due to games or rough-and-tumble in this process while they cannot pay attention to every child at the same time. Injuries occurred in the field mainly due to the lack of protective measures for minors and parents' attention.

Some studies show that open globe injury is much more prevalent than closed globe injury [30, 35], and the results were consistent in this study (70.5%). In all types of injury, rupture of the globe and penetrating injury are the two types with the largest number of minor victims. The cause of ocular trauma is closely related to the type of injury. Minors, especially younger children, are more prone to eye injury by writing, such as pencils and pens, which are very common causes of eye injuries in children [36]. So, the number of cases of cutting and stabbing with sharp instruments is also more common. Of note, traffic accidents are also a common cause of eye injuries among minors, which ranked second among all causes in this study, such as those caused by car safety bags can lead to serious eye injuries. One study showed that airbag-associated ocular trauma may necessitate intraocular surgery, may result in permanent visual loss, and may cause endothelial cell loss in pediatric patients [37]. Wearing seat belts and sitting in safety seats for minors are a very necessary precaution.

In some part of regions of China, children after injury cannot be transmitted to hospitals timely for economic reasons, road reasons, parents' perception, etc. Some studies have also focused on the time between injury and treatment [38]. One report noted that the average time (range) between the accident and seeking medical care was 17.4 hours (10 minutes to 14 days) [25], and in this study, we found that the average time interval between the occurrence of eye trauma and hospitalization of juveniles was 1 day. Based on clinical experience and publications [38], timely treatment can bring about a relatively good prognosis. China is a country with a large population, and the higher the hospital level, the more the number of patients, which makes many children in remote areas wait for a long time after an injury and lose the optimal timing for treatment. In this study, evisceration surgery was implemented in 37 cases, and even though the evisceration procedure was safe and effective [39], it can also cause great harm to children, so the prevention measurement and popular consciousness are very important and urgently needed to improve in the Chinese population.

Measuring VA and IOP in minor injuries is a formidable work, and nearly half of the data are unavailable in these patients. Many reports emphasized that different influencing factors (such as initial age and type of trauma) will cause different degrees of visual impairment in patients but ultimately will lead to poor vision of patients [40, 41]. In our study, the VA at discharge is better than that after the injury in general and the former will seriously affect the consequence at discharge if it is CF or below. So, whether the VA can be measured after the injury is an important factor for the outcome of injured eyes in minors in this study. With regard to IOP, data from this study showed that it could be effectively improved at discharge following treatment. Among all factors included in the analysis, younger age and normal IOP after injury had the greatest positive influence on the ocular condition at discharge.

Researchers around the world have called for increased awareness of eye injury protection among minors, especially recommendations for parents [28, 42], and some have made specific recommendations. The following strategies are aimed at reducing the incidence of eye trauma to fall into three main categories: legislation and policies, education, and personal eye protection [43]. One study suggests that French legislation should be stricter about the sale of any airsoft gun to children under 18 years old, and parents must repeat educational warnings to their children handling sharp objects [44]. Most eye injuries are preventable [25], for up to 90% of all ocular trauma is thought to be preventable through a combination of use of protective eyewear and education of supervising adults [45]. However, most patients do not take required measures or have lack of awareness of protection. Therefore, increasing awareness of the serious nature of ocular injuries and the study of the risk factors will help develop a comprehensive plan for educating both parents and children to minimize preventable eye injury sequelae.

Based on the actual situation of Chinese residents, this study puts forward the following feasible suggestions: (1) establish a green channel for minor trauma to ensure that patients can be salvaged by an experienced doctor in time; (2) compulsory wear of uniform goggles for physical activities (such as ball games) in schools; (3) government departments issue regulations to prohibit the sale of shooting and recreational articles with sharp edges to minors; (4) community workers are responsible for the regular implementation of children's eye injury protection courses.

The data in this study were all records of hospitalized cases of ocular trauma, and we checked records item by item. In addition, as mentioned before, our target hospital is large grade A hospitals in mainland China, so the cases admitted are generally severe eye injuries, which may lead to an overestimate of the proportion of open globe injury. The data of this study were based on the records of 30 hospitals in mainland China, which cannot fully represent the incidence of eye trauma among minors in China. In the future, a larger sample size and more in-depth studies, and eye injury registry network should be carried out and established.

In mainland China, the incidence rate of minor eye trauma is relatively high, mainly in young male children, and the main injury types are penetrating and rupture. Home and public buildings are the most common places for eye injuries among minors, so prevention strategies should be focused on parents' education and protective measures indoors. As a prognostic index, VA discharge will be relatively well if it is CF or higher after injury. Above all, minors should be provided with convenience in the treatment procedure to ensure the timely and effective treatments.

## 4. Additional Points

According to http://www.360baike.com, the geographical regionalization in China including North China (Beijing, Tianjin, Hebei, Shanxi, and Inner Mongolia Autonomous Region), East China (Shanghai, Jiangsu, Zhejiang, Anhui, Fujian, Jiangxi, Shandong, and Taiwan), South China (Guangdong, Guangxi Autonomous Region, Hainan, Hong Kong Special Administrative Region, and Macau Special Administrative Region), Central China (Henan, Hubei, and Hunan), Southwest China (Chongqing, Sichuan, Guizhou, Yunnan, and Tibet Autonomous Region), Northwest China (Shaanxi, Gansu, Qinghai, Ningxia Autonomous Region, and Xinjiang Autonomous Region), Northeast China (Liaoning, Jilin, and Heilongjiang), and the Inner Mongolia Autonomous Region was included in Northwest China, for most of the patients of the Affiliated Hospital of Inner Mongolia Medical University are coming from the west of Inner Mongolia.

## Figures and Tables

**Table 1:** Distribution of cases in boys and girls in different regions of China from 2019 to 2020.

Regions	Boys (*n* = 1398) (%)	Girls (*n* = 575) (%)	Total (*n* = 1973) (%)
Northeast	14 (1.0)	4 (0.7)	18 (0.9)
North	297 (21.2)	103 (17.9)	400 (20.3)
East	262 (18.7)	129 (22.4)	391 (19.8)
South	29 (2.1)	8 (1.4)	37 (1.9)
Central	416 (29.8)	166 (28.9)	582 (29.5)
Northwest	154 (11.0)	71 (12.3)	225 (11.4)
Southwest	226 (16.2)	94 (16.3)	320 (16.2)

**Table 2:** Gender distribution of trauma cases in different age groups from 2019 to 2020.

Age (years)	Boys (*n* = 1398) (%)	Girls (*n* = 575) (%)	Total (*n* = 1973) (%)
0–3	269 (19.2)	173 (30.1)	442 (22.4)
4–6	439 (31.4)	182 (31.7)	621 (31.5)
7–9	397 (28.4)	121 (21.0)	518 (26.3)
10–12	187 (13.4)	75 (13.0)	262 (13.3)
13–14	106 (7.6)	24 (4.2)	130 (6.6)

## Data Availability

The data used to support the findings of this study are available from the corresponding author upon request.
